# Traction test of temporary dental cements

**DOI:** 10.4317/jced.53732

**Published:** 2017-04-01

**Authors:** Juan-Luis Román-Rodríguez, Diego Millan-Martínez, Antonio Fons-Font, Rubén Agustín-Panadero, Lucía Fernández-Estevan

**Affiliations:** 1 Doctor in Dentistry (DDS; PhD). Master Buccofacial Prosthetics (M.Sc). Associate Lecturer, Department of Dental medicine, Prosthodontic and Occlusion Teaching Unit, University of Valencia General Studies (UVGS), Spain; 2 Lecturer in Prosthodontics, UVGS, Spain; 3 Doctor in Dentistry (DDS; PhD). Assistant Lecturer, Department of Dental medicine, Prosthodontic and Occlusion Teaching Unit, UVGS, Spain; 4 Doctor in Dentistry (DDS; PhD). Master Buccofacial Prosthetics (M.Sc). Associate Lecturer, Department of Dental medicine, Prosthodontic and Occlusion Teaching Unit , UVGS, Spain; 5 Doctor in Dentistry (DDS; PhD). Master Buccofacial Prosthetics (M.Sc). Associate Lecturer, De-partment of Dental medicine, Prosthodontic and Occlusion Teaching Unit, UVGS, Spain

## Abstract

**Background:**

Classic self-curing temporary cements obstruct the translucence of provisional restorations. New dual-cure esthetic temporary cements need investigation and comparison with classic cements to ensure that they are equally retentive and provide adequate translucence. The objective is to analyze by means of traction testing in a *in vitro* study the retention of five temporary cements.

**Material and Methods:**

Ten molars were prepared and ten provisional resin restorations were fabricated using CAD-CAM technology (n=10). Five temporary cements were selected: self-curing temporary cements, Dycal (D), Temp Bond (TB), Temp Bond Non Eugenol (TBNE); dual-curing esthetic cements Temp Bond Clear (TBC) and Telio CS link (TE). Each sample underwent traction testing, both with thermocycling (190 cycles at 5-55º) and without thermocycling.

**Results:**

TE and TBC obtained the highest traction resistance values. Thermocycling reduced the resistance of all cements except TBC.

**Conclusions:**

The dual-cure esthetic cements tested provided optimum outcomes for bonding provisional restorations.

** Key words:**Temporary dental cements, cements resistance.

## Introduction

Cementation is defined as the process of bonding a prosthetic element to a substrate with cement (1). Two types of cement are used in dentistry: definitive and temporary. The latter is used to bond a provisional prosthesis, to bond a definitive prosthesis temporarily, or to bond a prosthesis onto an implant. Both types of cement must provide adequate prosthetic retention and provide an effective seal between the post and the restoration; the seal must not suffer any changes as a result of the temperature changes in the oral cavity. It is also desirable that when a restoration is debonded, the cement stays on the restoration rather than on the tooth, as it clinically simpler and easier to remove the remaining cement extraorally.

Normally, temporary cements are classified according to their composition but they can also be classified according to their setting mechanism (1). Self-curing cements are those that consist of a base and a catalyzer, which set when they are mixed. They usually contain calcium hydroxide or zinc oxide. With dual-cure temporary cements, setting is activated by light; these are translucent resins and so considered esthetic.

1. To analyze the retention of five temporary cements by means of traction testing.

2. To assess the influence of thermocycling on temporary cements.

3. To determine the localization of remaining cement after traction testing/debonding.

## Material and Methods

This work is an *in vitro* study, each sample underwent traction testing, divided in thermocycling (190 cycles at 5-55º) and without thermocycling. This study has been carried out in Department of Dental Medicine. University of Valencia, Spain.

A metal model was fabricated to simulate a dental stump of standard dimensions as used in other studies (2). The stump was duplicated in plaster and then scanned using Cerec inLab® software (Sirona Dental Systems, NY, USA).

Meanwhile, ten human molars (extracted within the previous 3 months and conserved in physiological serum (3) were set in blocks of plaster shaped to fit the milling machine, which was then used to prepare the teeth following the previously scanned tooth stump model. In this way, 10 human molars were prepared exactly replicating the scanned tooth.

A coping was designed as provisional restoration, shaped to match the milled molars. The coping included a ‘handle’ to facilitate axial traction. Ten copings were milled from blocks of cross-linked polymethylmethacrylate (PMMA) (Telio CAD®, Ivoclar Vivadent, Schaan, Liechtenstein) for fabricating long-term provisional prostheses using CAD-CM techniques. A coping was randomly assigned to each molar.

Five temporary cements were tested, ( Table 1).

**Table 1:**
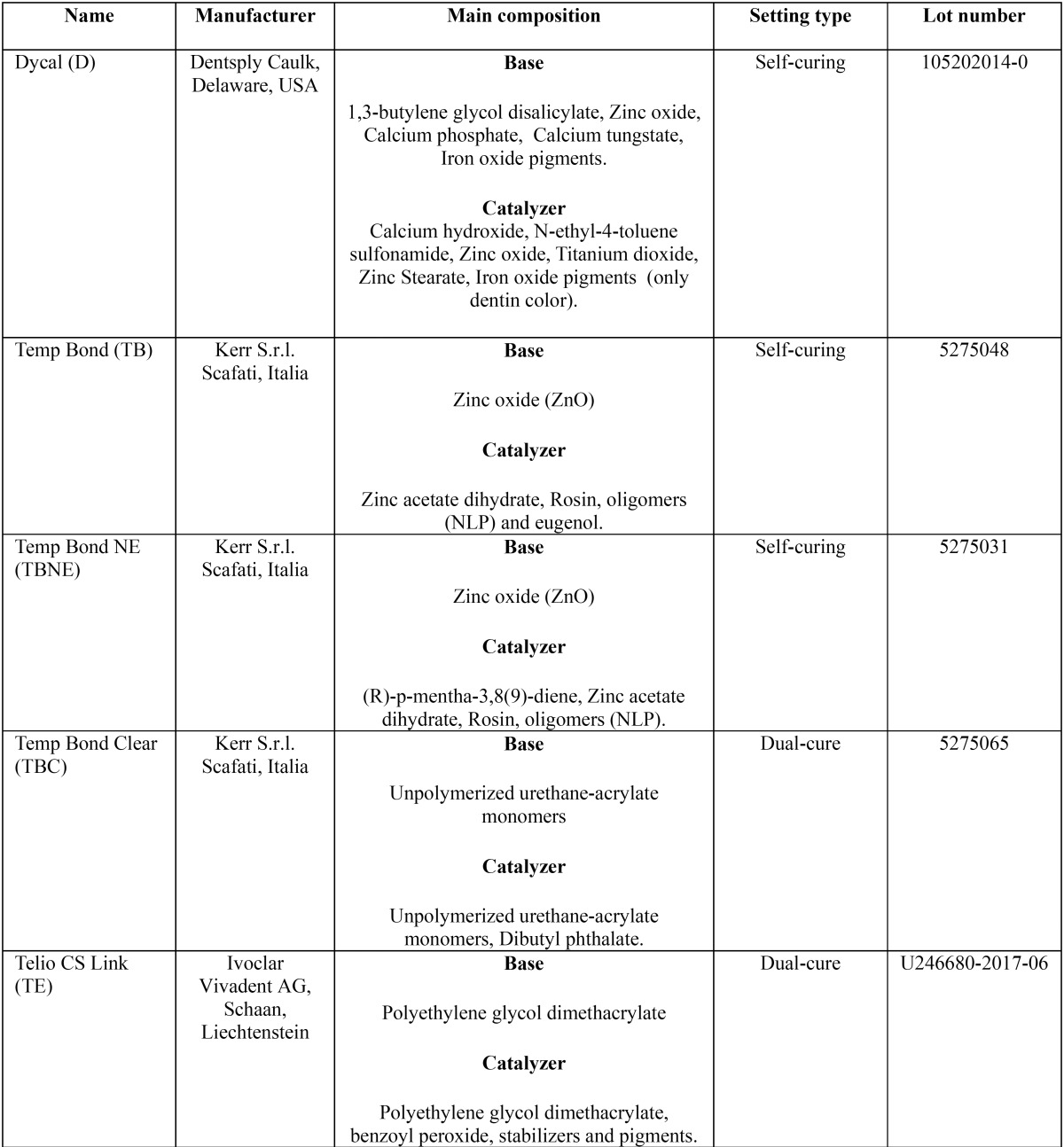
Cements tested in the study.

All ten copings were cemented with all five cements (five study groups, n=10). The five groups underwent traction testing (Instron 4804). After each test, both the molars and copings were cleaned first with a scalpel and then with a prophylaxis brush and abrasive dental paste (Détartine®, Septodont, Saint-Maur-des-Fossés, France) before proceeding to the next cement. When the five groups had been tested, the traction tests were repeated first subjecting the samples to thermocycling (190 cycles at 5-55º; Thermocycling TC-3, SD Mechatronik, Feldkirchen-Westerham, GERMANY), equivalent to approximately 7 days, the average time that a cemented temporary restoration remains in the mouth.

Statistical analysis was performed using SPSS-Windows® software (Statistical Package for the Social Sciences. SPSS Inc. Chicago, Illinois, USA), importing data from a single Microsoft® Excel spreadsheet. Descriptive and bivariate analyses were performed, applying the Pearson χ², Kruskal-Wallis and Mann-Whitney. The significance level established for all bivariate analysis was 1%, any *p*-value below 0.01 indicating a statistically significant difference.

## Results

**Figure 1:**
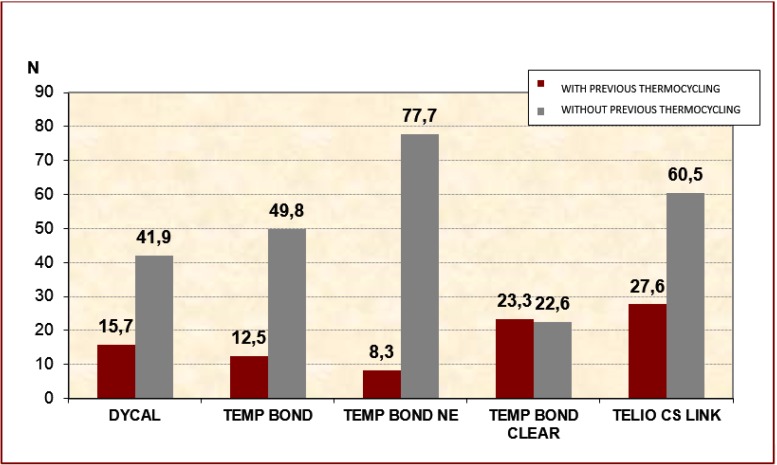
Mean resistance values for each cement, with or without thermocycling.

Temp Bond NE and Telio CS Link obtained the best initial retention values. After thermocycling, the retention values of all cements were seen to decrease, with the single exception of Temp Bond Clear. After thermocycling, Telio CS Link obtained the best retention, followed by Temp Bond Clear, (Fig. 1).

**Figure 2:**
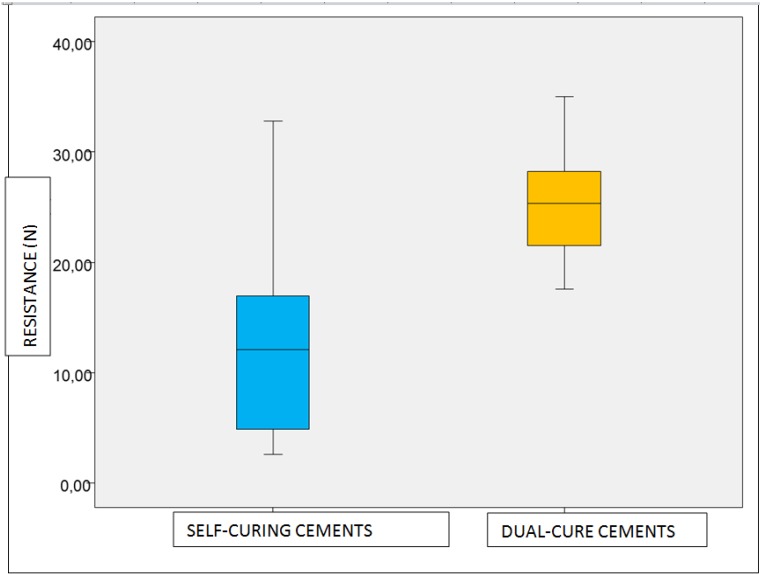
Box-plot of traction resistance after thermocycling for both types of temporary cements.

When the cements were grouped according to setting mechanisms, dual-cure cements presented the highest resistance values after thermocycling compared with classic self-curing cements, (Fig. 2).

**Figure 3:**
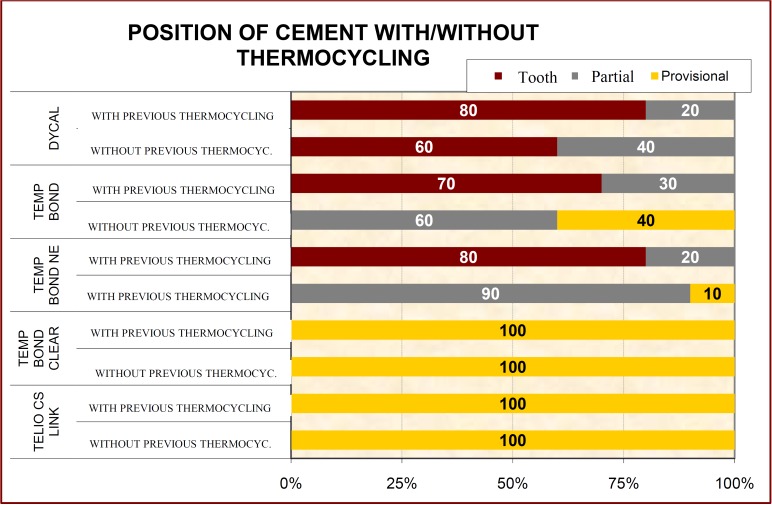
Localization of cement remains (on either restoration, tooth, or both [partial]) after debonding deriving from traction testing.

For dual-cure cements, the cement remaining after debonding was found on the restoration, while self-curing cements always remained on the tooth, (Fig. 3).

## Discussion

Few studies have investigated the retention of temporary cements, and the bibliography that does exist is scant, heterogeneous, and disparate. Each study uses a different methodology, particularly regarding the preparation of specimens, and while they provide an overview of general tendencies, it remains impossible to make a precise comparison of the results (4,5). The authors believe that the preparation method used in the present study proved both innovative and efficient, as it produced teeth of identical dimensions by means of reproducible CAD- CAM technology. This method will be the subject of a further article.

The results confirmed the considerable influence of thermocycling on retention values in all cases with a single exception - Temp Bond Clear. After thermocycling, the dual-cure cements obtained higher retention values, which shows that these cements are indicated in cases with short or convergent tooth stumps, when restoration retention may be compromised (1). The present results coincide with data obtained by Lawson in a study of self-curing and dual-cure cements that included thermocycling (1). Dual-cure cements are indicated for anterior teeth whenever the definitive restoration involves partial or complete coverage because of their translucency, retention capacity, and the fact that there is no eugenol in their composition. Non-eugenol cements will not compromise adhesion providing the definitive cementation is carried out with composite resins (6,7). Self-curing cements are indicated both in the posterior sector, in the anterior sector when it is to be restored with metal-ceramic restorations; in these cases cements with eugenol can be used as adhesive cementation of the definitive prosthesis is not a requirement (7).

Lepe *et al.* (8) did not find differences between Dycal, Temp Bond and Temp Bond NE, while Fernandes *et al.* found that Dycal achieved better retention, as in the present study (9). Rego *et al.* (10) obtained lower retention with Temp Bond NE, as did the present study.

It is important to bear in mind that if the immediate dentin sealing technique (IDS) is to be used, then it is recommended that dual-cure cements be avoided, as the tooth stump is protected with a resin after preparation and there will be a risk of union between the resin and the temporary cement (11,12).

The localization of temporary cement after debonding showed differences between the two types of cement, with more cement remaining on the restoration when dual-cure cements were used. It is better for cement to remain on the prosthesis rather than the tooth, as it can be cleaned extraorally, which saves inconvenience for the patient and avoids obliterating the dentinal tubules.

Within the limitations of this *in vitro* study, it may be concluded that:

1. Temporary dual-cure cements (Telio CS Link >Temp Bond Clear) obtain better retention than self-curing cements.

2. Thermocycling reduces retention of all the cements tested, with the exception of TBC.

3. After debonding, dual-cure cements remain on the restoration, while self-curing cements remain on the teeth.
